# Efficacy and safety of peptide receptor radionuclide therapy in advanced radioiodine-refractory differentiated thyroid cancer and metastatic medullary thyroid cancer: a systematic review

**DOI:** 10.1186/s12885-021-08257-x

**Published:** 2021-05-20

**Authors:** Zohreh Maghsoomi, Zahra Emami, Ramin Malboosbaf, Mojtaba Malek, Mohammad E. Khamseh

**Affiliations:** 1 Endocrine Research Center, Institute of Endocrinology and Metabolism, Iran University of Medical Science (IUMS), No. 10, Firoozeh St, Vali-asr Ave, Vali-asr Sq, Tehran, 1593716615 Iran; 2 Research Center for Prevention of Cardiovascular Disease, Institute of Endocrinology and Metabolism, Iran University of Medical Sciences (IUMS), Tehran, Iran

**Keywords:** Peptide receptor radionuclide therapy, Radioiodine refractory-differentiated thyroid Cancer, Medullary thyroid carcinoma, Papillary thyroid carcinoma, Yttrium-90, 177Lu-DOTATATE, Indium-111, Systematic review

## Abstract

**Background:**

It has been shown that a subgroup of patients with differentiated thyroid cancer (DTC) and medullary thyroid carcinoma (MTC) would progress to advanced stages of thyroid cancer. Therefore, the present study was done to systematically review available evidence in order to investigate efficacy and safety of peptide receptor radionuclide therapy **(**PRRT) in the patients with advanced radioiodine refractory differentiated thyroid cancer (RR-DTC) and metastatic MTC.

**Methods:**

For this purpose, relevant studies investigated safety and efficacy of PRRT in the patients with advanced RR-DTC and metastatic MTC were identified by searching Medline (Pubmed, Ovid, and Ebsco), Scopus, Embase, Web of Science, and Cochrane Library databases (from database inception to March 24, 2021). The review was performed according to the preferred reporting items for systematic reviews and meta-analyses (PRISMA) statement. Searching was done independently by two investigators. Two researchers independently extracted the data and any disagreement was adjudicated by consensus. Quality of the studies was assessed using the tool of case reports/series in systematic reviews.

**Results:**

Among 2284 related papers, 41 papers met the inclusion criteria. A total of 157 patients with RR-DTC were treated with PPRT. Biochemical and objective responses (partial and complete) were observed in 25.3 and 10.5% of patients, respectively. Among 220 patients with metastatic MTC, biochemical and objective responses were observed in 37.2 and 10.6% of the patients, respectively.

Forty-six deaths were reported in 95 patients with advanced RR-DTC. In addition, 63 deaths were observed in 144 patients with metastatic MTC. Major side effects were reported in 124 patients treated with ^90^Y -based agent. In the patients treated with 177Lu-DOTA-TATE and 111In-Octreotide, mild and transient hematologic or renal complications were reported.

**Conclusion:**

Findings of the study revealed that in the absence of the established treatment for the patients with RR-DTC and metastatic MTC, PRRT could be effective with few adverse events.

**Trial registration:**

PROSPERO registration number: CRD42019125245.

**Supplementary Information:**

The online version contains supplementary material available at 10.1186/s12885-021-08257-x.

## Background

Thyroid cancer is the most common endocrine malignancy and its incidence has increased by 4.4% per year during 20072011 [1, 2]. Differentiated thyroid cancer (DTC), is the most frequent subtype of thyroid cancer accounting for 8595% of the cases [3, 4]. Medullary thyroid cancer (MTC) originating from parafollicular or C cells of the thyroid gland accounts for approximately 5% of all thyroid cancer cases [5].

The standard of treatment for most patients with DTC includes thyroidectomy followed by radioiodine treatment. A 10-year overall survival rate of 8099% has been reported among these patients [6]. However, in spite of highly effective treatment strategies, there is a chance of recurrence in 20% of the subjects. Radioactive iodine plays a major role in diagnosis and treatment of recurrent disease [7]. However, some thyroid cancers are resistant to radioiodine despite the elevated level of thyroglobulin [8]. Radioiodine refractory-DTC (RR-DTC) has shown aggressive clinical behavior and a 10-year survival rate of 10% [9, 10]. Surgery and external beam radiation therapy can be used to manage local disease but not in case of widespread metastases. Moreover, chemotherapeutic agents have shown limited efficacy with considerable side effects [11, 12].

MTC is inherently non-sensitive to radioactive iodine. Hence, its management is more difficult and its prognosis is worse than DTC [7]. The overall survival rate is between 75 and 85% during 10years for individuals with MTC [13]. In spite of aggressive surgical treatment, there is almost a 50% of chance for persistent or recurrent disease, with deleterious effects on quality of life and the reduced 10-year survival rate by 40% [7, 13]. Reoperation, embolization, and perhaps radiotherapy could improve outcomes [14]. Meanwhile, response to conventional chemotherapy is limited with life-threatening toxicity [7]. Currently, other therapeutic options are scarce and not widely available.

There are few alternative treatments in the patients with advanced RR-DTC. Somatostatin receptor (SSTR) expression on cell surface of neuroendocrine and thyroid tumors regulates cell proliferation [15]. Targeting SSTR with radiotracer in peptide receptor radionuclide therapy (PRRT) can induce tumor cell death. Overexpression of somatostatin receptor subtypes on surface of cells is required for PRRT and therefore, tumor remission can be predicted based on the results of scintigraphy on somatostatin receptor. Thus, PRRT could be a therapeutic option based on scintigraphy results of somatostatin receptor. It has been used previously for treatment of metastatic neuroendocrine tumor and advanced pheochromocytomas and paragangliomas with high efficacy, tolerability, and low toxicity [16, 17].

Accordingly, the present study was conducted to systematically review available evidence in order to investigate efficacy and safety of PRRT in the patients with advanced RR-DTC and metastatic MTC.

## Methods

### Search strategy and selection criteria

A systematic review was performed on the published works to investigate safety and efficacy of PRRT in the patients with advanced RR-DTC and metastatic MTC, according to the preferred reporting items for systematic reviews and meta-analyses (PRISMA) statement [18]. The study was registered before completing formal screening of search results (PROSPERO registration number: CRD42019125245).

### Eligibility criteria

All the original studies containing data related to PRRT were considered eligible to be included in the review study. Exclusion criteria were irrelevant papers (based on screening of titles and abstracts), papers with insufficient data available, duplications, and review papers. All the eligible studies were included to assess efficacy, and/or safety of PRRT.

### Study identification

For this systematic review, the Cochrane Central Register of Controlled Trials (Central), Medline (PubMed, Ovid, and Ebsco), Scopus, and Embase databases were searched (from database inception to March 24, 2021). Search terms for English-language publications included: peptide receptor radionuclide therapy, PRRT, radionuclide therapy, radiolabeled somatostatin analogues, thyroid cancer, thyroid carcinoma, thyroid neoplasm, differentiated thyroid cancer, differentiated thyroid carcinoma, differentiated thyroid neoplasm, medullary thyroid cancer, medullary thyroid carcinoma, and medullary thyroid neoplasm. Details regarding the search strategy are provided in the Supplementary Table1.

The first search was done independently by two investigators (ZE and ZM). Also, a complete updated search was performed on all databases available and new studies (if any exist) were identified to assess the details and incorporate findings in this review. The snowballing techniques were used to complete the search by screening reference lists of the included papers for relevant studies. Also, registry of prospective studies with accessible results was searched. Two authors (RM, ZM) independently determined studies that should be evaluated further by scanning the title, abstract, or both based on the inclusion/exclusion criteria, the reviewers were blinded to names of the journals and authors. All the potentially relevant papers as full texts were assessed and any disagreements were resolved by consensus or by arbitration of two experts (MK and MM). In case of duplicates or multiple publications of a primary study, yield of information was enhanced by collating all available data and using the most complete data set aggregated across all the known publications.

### Data collection and management

Two reviewers (RM and ZM) independently extracted the data from the included trials and any disagreement was adjudicated by consensus or by arbitration of other reviewers (MK and MM). Published reports were obtained for every study, and standard information was extracted in a spreadsheet. The following data were extracted: authors name; year of publication; country where the study was performed; number of participants, sex and age of the participants; tumor classification, site of metastases; prior treatments (cumulative radioiodine in RR-DTC); cumulative activity (GBq) of PRRT**;** response to treatment criteria; time to progression (TTP); follow-up duration; response to treatment; complications (major/minor); mortality rate; and time to death.

Biochemical response was defined in the patients with DTC based on serum thyroglobulin (Tg) level and in the patients with MTC, it was defined based on serum calcitonin and carcino- embryonic antigen (CEA) levels. Different criteria were used to evaluate radiological responses to treatment, namely world health organization (WHO) criteria, response evaluation criteria in solid tumors (RECIST) criteria, and southwest oncology group (SWOG) criteria [19]. Moreover, the European organization for research and treatment of cancer (EORTC) has classified metabolic response to treatment based on the maximum standardized uptake value (SUVmax) [20].

For further analysis, proportions of complete and partial radiologic response were integrated as objective response.

Occurrence of adverse events was evaluated using common terminology criteria for adverse events (CTCAE) [21]. Two reviewers (RM and ZM) independently assessed methodological quality of the included studies using the tool of systematic reviews [22], and any disagreement was resolved by consensus.

## Results

**Fig. 1:**
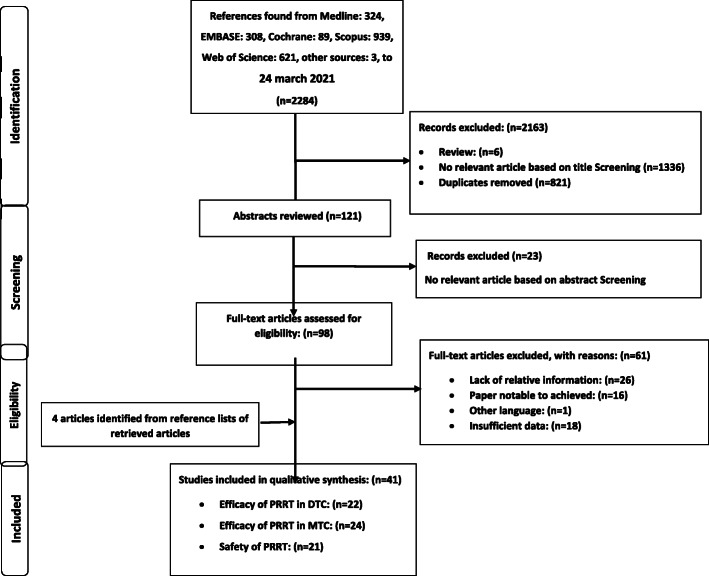
Flow chart of literature search and article selection

Search on the literature led to identification of 2284 publications, of which 98 papers were reviewed in full text (Fig.1. shows flow chart of literature search and paper selection). The risk of bias of the included studies was low (Supplementary Table2). Inter-reviewers agreement was excellent for the selected papers (Cohens test =0.96). Among 41 publications met the inclusion criteria, 12 papers were retrospective in terms of design; 19 papers were prospective studies and remaining 10 papers were case reports. Tables1 and 2 summarize characteristics of the included studies assessing efficacy of PRRT in the patients with advanced RR-DTC, and metastatic MTC, respectively. Data regarding safety of PRRT are presented in Table3. Cumulative activity of PRRT ranged between 0.92583.2GBq. For ^90^Y -based agent, most of the studies had used this agent with an administered activity ranging from 0.925 to 5.9GBq per cycle usually up to 4cycles. For 177Lu-DOTA-TATE*,* the administered activity rate was between 5.57.7GBq per cycle usually up to 4cycles. In terms of follow-up duration, in the patients with advanced RR-DTC, it was between 1 and 99months after commencement of PRRT (median: 12months). It was between 1 and 144months (median: 17months) in the patients with metastatic MTC. Death was recorded in 109 patients. Time to death varied from 1 to 63months (median: 11months). It should be noted that more than one criterion was used to evaluate efficacy of PRRT, and some patients did not complete their full course of treatment.

**Table 1:** Efficacy of peptide receptor radionuclide therapy (PRRT) in patients with advanced RR-DTC^a^

Reference (Publish Year)	Country	N	Sex	Age (year)	Tumor Classification	Site of metastasis	Prior treatments (Iodine cumulative activity GBq (med))	Ligand (Radionuclide Chelator Peptide)	Cumulative activity (GBq)	Response criteria	TTP in SD (month)	Follow-Up duration median: (months)	Response
Czepczynski R et al. (2014) [15]	Poland	6	F/M: (9/2)	Median: 65 (4781)	3FTC, 3HCTC	B/Lu/M	TT/ND/EBR/RIT (3.1)	90Y-DOTATOC	3.7_14.8	Biochemical	NA	21 (2_68)	4PD, 1PR, 1SD
										RECIST			2PD, 2SD. 1PR
Versari A et al. (2014) [23]	Italy	11	F/M	Median: 59 (1978)	5PTC, 1Oxiphilic, 3FTC, 2Insular	Li/B/Lu	TT/ND/RIT (5.5533.3 (12.95))	90Y-DOTATOC	4.329_17.95	Biochemical	NA	7.75 (3.5_11.5)	11SD
										RECIST			2PR, 4SD, 4PD, 1NA
										EORTC			2PR, 5SD, 4PD
Iten F et al. (2009) [24]	Switzerland	24	F/M: (12/12)	Median: 58.8 (40.580.6)	17FTC, 5PTC, 2No specified	NA	TT/ND/RIT	90Y-DOTATOC	5.630.3	Biochemical	NA	16.8 (1.8_99.1)	7PR, 17PD
Gabriel M et al. (2004) [25]	Austria	5	F/M: (2/3)	Median:59 (51_72)	3FTC, 2PTC	B/Lu/M	TT/ND/RIT (9.25_29.91 (18.87))	90Y-DOTATOC	5.55_7.4	NA	5	NA	5SD
Gorges R et al. (2001) [26]	Germany	3	F/M: (2/1)	Median: 68 (51_72)	1papillary-oxyphilic, 1follicular-oxyphilic, 1Hrthle cell carcinoma	Li/B/Lu/M	TT/ND/EBR/RIT	90Y-DOTATOC	1.7_9.62	Biochemical	NA	20 (16_31)	2SD,1PD
										RECIST	NA		1SD, 2PD
Waldherr C et al. (2001) [27]	Switzerland	7	F/M: (4/3)	Median:60 (44_74)	4PTC, 3FTC	NA	TT/ND/EBR/RIT/C/EN	90Y-DOTATOC	1.7_14.8	WHO	8	15 (1_31)	2SD, 5PD
Virgolini I et al. (2002) [28]	UK	25	NA	NA	NA	NA	NA	90Y-DOTA-Lanerotide	0 .925_7.06	WHO	NA	36	3PR, 11SD, 11PD
Traub-Weidinger T et al. (2011) [29]	Austria	4	NA	Median:66	1FTC, 1HCTC	B/Lu	TT/ND/EBR/RIT	90Y-DOTATOC	7.2_7.4	Imaging	NA	(22_27)	4PD
					1FTC, 1PTC	Lu		90Y-DOTA-Lanerotide	1.85_3.7	Imaging	NA	(4_12)	PD
Basu. S et al. (2020) [30]	India	8	M/F: (5/3)	57_83	1FTC	B/Lu/M	TT/ND/RIT	177Lu-DOTATATE	5.5_25.4	Biochemical	NA	34(7_52)	5PD, 3PR
										RECIST			6PD, 2SD
Cinkir, H. Y et al. (2020) [31]	Turkey	4	M/F: (3/1)	Median: 64 (49,67)	1FTC, 3PTC	Lu/B	TT/ND/RIT/C	177Lu-DOTATATE	14.8_30.8	EORTC	5.5 (1.7_9.4)	13.8 (4.0_23.7)	1PD, 2SD, 1PR
Roll. W et al. (2018) [32]	Germany	5	M/F: (4/1)	Median: 75 (62_89)	3FTC, 1PTC, 1HCTC	NA	TT/ND/EBR/RIT	177Lu-DOTATATE	Mean: 7.00.7	Biochemical	NA	6 (3_9)	1FTC PR, 4PD
										RECIST			2PD, 2SD, 1PR
										EORTC			3PD, 2SD
Olivn-Sasot. P et al. (2017) [33]	Spain	1	F	69	FTC	B/Li	TT/ND/C/RIT (10.4)	177Lu-DOTATATE	2.6	Biochemical	NA	6	PR
Elboa, U et al. (2016) [34]	Turkey	1	M	64	PTC (tall cell variant)	B/ Lu/M	TT/ND/RIT (27.75)	177Lu-DOTATATE	7.4	Biochemical	NA	After second cycle	PR
Jois B et al. (2014) [35]	India	1	NA	NA	PTC	Lu	TT/ND/EBR/RIT (NA)	177Lu-DOTATATE	7.4	Biochemical	NA	3	PR
										Imaging			SD
Teunissen JJ et al. (2005) [36]	Netherlands	5	F/M	Median: 52 (52_74)	3HCTC, 1FTC, 1PTC	B/Lu	TT/ND/EBR/C/RIT (1.9_16.7 (12.9))	177Lu-DOTATATE	22.430.1	Biochemical	Median: 22(4_43)	(4_48)	2PD, 3PR
										WHO			2SD, 1PD, 1PR, 1MRe
Parihar AS et al. (2018) [37]	India	1	F	54	PTC	B/Lu	TT/ND/RIT (18.5)	177Lu-DOTA-RGD2	5.5	RECIST	NA	4	SD
Campenni A et al. (2015) [38]	Italy	1	M	70	PTC	Lu	TT/ND/RIT (3.7)	177Lu-DOTATOC	7.77	Biochemical	5	5	PR
										RECIST			SD
Valkema R et al. (2002) [39]	Netherlands	5	NA	Median:70.8 (57.3_76.1)	4PTC, 1FTC	Lu	TT/ND/BT/RIT	1111n-Octerotide	29.51_83.2	Biochemical	NA	15.8 (15_16.6)	1SD, 1PD, 1PR, 2NA
										SWOG		15.8 (13.2_28.2)	1SD, 4PD
Krenning E et al. (1999) [40]	Netherlands	1	NA	NA	PTC	NA	NA	1111n-Octerotide	20_75	imaging	NA	24	1SD
Stokkel MP et al. (2004) [41]	Netherlands	11	F/M: (7/4)	Median:67 (4469)	6PTC, 5FTC	Li/B/Lu/M	TT/ND/EBR/C/Emb/RIT	111In-DTPA-Octreotide	14.3_33.1	Biochemical	NA	12 (1_12)	7SD, 3PD, 1NA
										Imaging			4SD, 5PD, 2NA
Budiawan H et al. (2013) [6]	Germany	7	F/M: (5/2)	Median: 64.5 (26_77)	4FTC,3HCTC	A/Li/Lu/B	TT/ND/EBR/C/RIT/LITT/REDIFF	90Y-DOTATATE and 177Lu-DOTATATE	NA	EORTC	NA	50.4 (34.866)	1SD, 5PD, 1PR
Scalorbi F et al. (2017) [42]	Italy	21	F/M: (13/8)	NA	NA	NA	TT/ND/RIT	NA	NA	EORTC	NA	NA	2PR, 9PD, 10SD

^a^Abbreviations: *NA* Not Available, *FTC* Follicular Thyroid Carcinoma, *RR-DTC* Radioiodine-Refractory Differentiated Thyroid Cancer, *PTC* Papillary Thyroid Carcinoma, *HCTC* Hurtle Cell Thyroid Carcinoma, *TTP* Time To Progression

Metastatic Site: *A* Adrenal, *Li* Liver, *Lu* Lung, *B* Bone, *M* Mediastinum

Prior treatments: *TT* Total Thyroidectomy, *ND* Node Dissection, *EBR* External Beam Radiation, *C* Chemotherapy, *I* Radioactive Iodine therapy, *BT* Biotherapy with octreotide, *LITT* Laser- induced thermotherapy, *REDIFF* Redifferentiation Using Roaccutane

Response: *CR* Complete Response, *PR* Partial Remission, *SD* Stable Disease, *PD* Progressive Disease, *MR* Minor Remission

**Table 2:** Efficacy of peptide receptor radionuclide therapy in patients with metastatic MTC^a^

Reference (Publish Year)	Country	N	Sex	Age	Site of metastasis	Prior treatment	Ligand (Radionuclide Chelator Peptide)	Cumulative activity (GBq)	Response criteria	TTP in SD (month)	Follow-Up duration (months)	Response
ksz M et al. (2014) [43]	Switzerland	1	NA	NA	NA	TT/ND/C	90Y-DOTA-TOC	5.65	Biochemical	NA	3	PD
									RECIST			PD
									EORTC			PD
Bertagna F et al. (2009) [44]	USA	1	M	74	B/M/ H	TT/ND/RF	90Y-DOTA-TOC	9.01	Biochemical	NA	7	SD
									RECIST	NA		SD
									WHO	NA		SD
									EORTC	NA		PR
Iten F et al. (2007) [45]	Switzerland	31	F/M: 10/21	Mean: 56.7 (24.076.9)	NA	TT/ND/C/EBR	90Y-DOTA-TOC	1.729.6	Biochemical	NA	15.7 (1.4_107)	9R, 22NR
Bodei L et al. (2004) [46]	Italy	21	F/M: 8/13	Median: 53 (3178)	Lu/Li/B/M	TT/ND/C/EBR/BT	90Y-DOTA-TOC	7.519.2	Biochemical	NA	40	3SD, 12PD, 5PR, 1CR
									SWOG	NA		12SD, 7PD, 2CR
Gao ZR et al. (2004) [47]	China	1	M	58	Lu/ M	NA	90Y-DOTA-TOC	3.33	Biochemical	6	10.5	PR
									WHO			SD
Waldherr C et al. (2001) [27]	Switzerland	12	F/M: 5/7	Median: 60 (24_72)	NA	TT/ND/C/EBR/BT/EN	90Y-DOTA-TOC	1.7_14.8	WHO	10 (3_14)	15 (1_31)	5SD, 7PD
Otte A et al. (1999) [48]	Switzerland	2	F	65	NA	NA	90Y-DOTA-TOC	9.25_9.62	WHO	NA	24	2SD
Bilgic, S et al. (2020) [49]	Turkey	19	F/M: 6/13	32_87	Lu/Li/B/M	TT/ND/C	177Lu-DOTATATE	6.5_52.3	Biochemical	NA	NA	7SD, 8PR, 4PD
									Imaging			15SD, 2PR, 2PD
Cinkir, H. Y et al. (2020) [31]	Turkey	3	M	Median: 53 (38,59)	Lu/B/M	TT/ND/C/EBR	177Lu-DOTATATE	14.8_44.4	EORTC	37.3 (17.6_56.9)	24.2 (0_48.8)	3SD
Parghane, R. V et al. (2020) [50]	India	43	F/M: 8/35	Median: 48 (25,80)	Lu/Li/B/M	TT/ND/C/EBR	177Lu-DOTATATE	5.55_33.3	Biochemical	24 (15.1_32.9)	26 (16.6_35.3)	5CR, 4SD, 13PR, 21PD
									RECIST			22SD, 4PR, 17PD
Makis W et al. (2015) [51]	Canada	2	NA	Median: 56.5 (38,75)	B/M	TT/ND	177Lu-DOTATATE	22.2	Biochemical	NA	9.5 (9,10)	1PR, 1PD
									WHO			2SD
Vaisman F et al. (2015) [52]	Brazil	7	NA	Median: 35.8 (20_54)	NA	NA	177Lu-DOTATATE	29.6	RECIST	NA	12	3PR, 3SD, 1PD
Soydal et al. (2014) [53]	Turkey	2	F/M	Median: 41	Lu/Li	TT/ND/EBR	177Lu-DOTATATE	29.6	RECIST	NA	6weeks After fourth cycle	2SD
Beukhof, C et al. (2019) [54]	Netherlands	10	F/M: 6/4	Median: 62 (1975)	NA	NA	177Lu-octreotide	27.8_29.6	Biochemical	8.4 (3.6_144)	16.88 (4.8_144)	3SD, 4PR, 3PD
									RECIST			4SD, 6PD
Mathew, D et al. (2018) [55]	India	2	NA	NA	NA	NA	177Lu-octreotide	NA	Imaging	NA	7weeks After last cycle	2SD
Pasieka JL et al. (2004) [56]	Canada	1	M	46	M	TT/ND/C	1111n-Octerotide	11.954	Biochemical	NA	9	PD
									SWOG			
Valkema R et al. (2002) [39]	Netherlands	5	NA	Median: 57.4 (27.7_77.4)	B/Lu	TT/ND/C/EBR/BT	1111n-Octerotide	25.14_87.28	Biochemical	NA	7.8 (2.76_26.8)	2SD, 3PD
									SWOG		7.8 (2.76_26.8)	3SD, 2PD
Caplin M et al. (2000) [57]	Poland	1	F	46	NA	NA	1111n-Octerotide	11.4	Biochemical	NA	NA	CR
Krenning E et al. (1999) [40]	Netherlands	3	NA	NA	NA	NA	1111n-Octerotide	NA	Imaging	NA	24	1SD, 2PD
Buscombe JR et al. (2003) [58]	UK	2	NA	Median: 52 (4658)	NA	NA	1111n-pentetreotide	25_75	RECIST	NA	27.5 (22,33)	2CR
Hayes AR et al. (2019) [59]	UK	9	NA	NA	NA	NA	90Y-DOTATATE and/or 177Lu-DOTATATE	NA	Biochemical	14 (820)	NA	6PR, 3PD
Puranik A et al. (2019) [60]	India	28	F/M: 14/14	Mean: 47.9 (26_72)	NA	TT/ND/C/EBR	90Y-DOTATATE and 177Lu-DOTATATE	NA	EORTC	NA	36	17SD
											72	5PR
											24	6PD
Budiawan H et al. (2013) [6]	Germany	7	F/M: 3/4	Median: 66.5 (21_68)	Lu/Li/B	TT/ND/EBR/	90Y-DOTATATE and 177Lu-DOTA-TATE	NA	EORTC	NA	50.4 (34.866)	4SD, 1PD, 1PR
Scalorbi F et al. (2017) [42]	Italy	7	F/M: (4/3)	NA	NA	NA	NA	NA	NA	NA	NA	5SD, 2PD

^a^Abbreviations: *NA* Not Available, *MTC* Medullary Thyroid Carcinoma, *TTP* Time To Progression

Metastatic Site: *A* Adrenal, *Li* Liver, *Lu* Lung, *B* Bone, *M* Mediastinum

Prior treatments: *TT* Total Thyroidectomy, *ND* Node Dissection, *EBR* External Beam Radiation, *C* Chemotherapy, *BT* Biotherapy with Octreotide, *LITT* Laser Induced Thermotherapy, *REDIFF* Redifferentiation Using Roaccutane

Response: *CR* Complete Response, *PR* Partial Remission, *SD* Stable Disease, *PD* Progressive Disease, *MR* Minor Remission

**Table 3:** Safety of Peptide Receptor Radionuclide Therapy in patients with Advanced RR-DTC & Metastatic MTC^a^

Reference	Kind of PRRT	Number & Kind of Tumor	Cumulative activity (GBq)	Hematologic Toxicity	Gastrointestinal& Hepatobiliary Toxicity	Genitourinary Toxicity	Others	Mortality (Median time to death since the first course of PRRT (months))
Bertagna F et al. [44]	90Y-DOTATOC	1MTC	9.01	None	None	None	None	1 (NA)
Bodei L et al. [46]	90Y-DOTATOC	21MTC	7.519.2	15	NA	None	NA	4 (NA)
Bodei L et al. [61]	90Y-DOTATOC	4MTC	3.8_19.2	None	None	None	None	None
Czepczynski R et al. [15]	90Y-DOTATOC	3FTC, 3HCTC	3.7_14.8	6	None	2	None	1 (63)
Gorges R et al. [26]	90Y-DOTATOC	1papillary-oxyphilic, 1follicular-oxyphilic, 1Hrthle cell carcinoma	1.7_9.62	3mild Lymphocytopenia	None	None	None	1 (16)
Iten F et al. [24]	90Y-DOTATOC	17FTC,5PTC, 2No specified	5.630.3	3Anemia, 3Transient Thrombocytopenia, 1Transient Leukopenia	4nausea	4permanent renal toxicity	None	11FTC, 4PTC, 2No (13.7)
Iten F et al. [45]	90Y-DOTATOC	31MTC	74.5	3Transient Leukopenia, 1Transient Thrombocytopenia	5Nausea	6	NA	22 (25.8)
Versari A et al. [23]	90Y-DOTATOC	5PTC, 1Oxiphilic, 3FTC, 2Insular	4.329_17.95	2Transient Anemia, 2Transient Leukopenia	4nausea, 1transient increase of transaminase	1permanent renal toxicity	2Asthenia	None
Waldherr C et al. [27]	90Y-DOTATOC	12MTC, 4PTC, 3FTC	1.7_14	6Anemia, 10Transient Lymphocytopenia	NA	NA	NA	1 (1)
Traub-Weidinger T et al. [29]	90Y-DOTATOC	1FTC, 1PTC	7.2_7.4	None	None	1renal toxicity	None	1 (22)
	90Y-DOTA-Lanerotide	1FTC, 1HCTC	1.85_3.7	2Transient Thrombocytopenia		None		2 (4_12)
Basu. S et al. [30]	177Lu-DOTATATE	8 DTC	5.5_25.4	None	None	1 transient	None	2 (7_12)
Beukhof, C et al. [54]	177Lu-Octreotate	10MTC		None	1Diarrhea	None	1Hemoptysis	7MTC, 1another cause
Cinkir, H. Y et al. [31]	177Lu-DOTATATE	3MTC, 3PTC, 1FTC	14.8_44.4	2Transient Anemia, 3Transient Leukopenia	None	None	None	1MTC,2PTC
Parghane, R. V et al. [50]	177Lu-DOTATATE	43MTC	5.55_33.3	1Transient	1Nausea	None	None	20
Teunissen Jj et al. [12]	177Lu-DOTATATE	3HCTC, 1FTC, 1PTC	22.430.1	NA	NA	NA	NA	1 (48), 1 (4)
Vaisman F et al. [52]	177Lu-DOTATATE	7MTC	NA	NA	NA	NA	1transient sexual dysfunction, 2mild hair Loss, 1hypersensitivity dermatologic lesions	2 (1/7 before the end of the protocol)
Valkema R et al. [39]	1111n-Octerotide	5MTC, 5DTC	NA	None	None	None	None	4MTC (11.22 (2.76_26.8)), 5DTC (15.8 (13.2_28.2))
Stokkel Mp et al. [41]	111In-DTPA-Octreotide	6PTC, 5FTC	14.3_33.1	1thrombocytopenia	None	None	None	1 (5), 2non related (1, 3)
Budiawan H et al. [6]	90Y-DOTATATE and 177Lu-DOTATATE	7MTC, 4FTC, 3HCTC	NA	8minor hematology, 5Anemia, 1Leukopenia	6transient increase of transaminase	5mild renal toxicity	None	1MTC (12), 2FTC (12), 1HCTC (24)

Abbreviations: *Na* Not Available, *RR-DTC* Radioiodine-Refractory Differentiated Thyroid Cancer, *FTC* Follicular Thyroid Carcinoma, *PTC* Papillary Thyroid Carcinoma, *HCTC* Hurtle Cell Thyroid Carcinoma, *MTC* Medullary Thyroid Carcinoma

### Efficacy of PRRT in RR-DTC

Overall, 157 patients with advanced RR-DTC were treated with PRRT. Based on biochemical response criteria, from 79 treated patients, 20 cases of partial response (PR), 22 cases of stable disease (SD), and 37 cases of persistent disease (PD) were determined. Out of 91 patients whose radiological response was assessed, 9 cases of PR, 39 cases of SD, and 43 cases of PD were recorded. Metabolic response was evaluated in 48 patients. Six cases of PR, 20 cases of SD, and 22 cases of PD were identified.

In 85 patients treated with ^90^Y -based agent; 44 patients were assessed based on biochemical response among whom 8 cases of PR, 14 cases of SD, and 22 cases of PD were observed. Seven cases of PR, 23 cases of SD, and 25 cases of PD were identified in 55 patients assessed based on radiological response. Moreover, 2 cases of PR, 5 cases of SD, and 4 cases of PD were reported in 11 patients assessed based on metabolic response.

In 26 patients treated with Lutetium-177 -based agent, 10 cases of PR, and 11 cases of PD showed biochemical response. Considering 20 patients assessed for radiological response, 2 cases of PR, 9 cases of SD, and 9 cases of PD were reported. Out of 9 patients assessed for metabolic response, 1 case of PR, 4 cases of SD, and 4 cases of PD were identified.

Moreover, in 18 patients treated with Indium-111, biochemical response was assessed in 14 patients. Two patients with PR, 8 cases with SD, and 4 cases with PD were reported. Seven SD cases and 9 PD cases were recorded based on radiological response in 16 patients.

Among 157 patients with RR-DTC, biochemical and objective responses (partial and complete) were observed in 25.3 and 10.5% of the patients, respectively.

### Efficacy of PRRT in metastatic MTC

In total, 220 patients with metastatic MTC were treated with PRRT. Based on biochemical response to the treatment in 145 patients, 7 cases of complete response (CR), 47 cases of PR, 20 cases of SD, and 71 cases of PD were recognized.

Radiologic response was evaluated among 134 patients. Four cases of CR, 9 cases of PR, 75 cases of SD, and 46 cases of PD were observed. Considering metabolic response among 46 patients, 7 cases of PR, 29 cases of SD, and 10 cases of PD were identified.

Sixty-nine patients were treated by ^90^Y-DOTATOC, 88 patients were treated with 177Lu-DOTA-TATE, and 12 patients were treated with 111_Indium -based agent. Type of treatment was unknown in other patients.

In 69 patients treated with ^90^Y-DOTATOC, 1 case of CR, 15 cases of PR, 4 cases of SD, and 35 cases of PD (based on biochemical response criteria in 55 patients) as well as 2 cases of CR, 21 cases of SD and 15 cases of PD (based on radiological response criteria in 38 patients) and 1 case of PR and 1 case of PD (based on metabolic response criteria in 2 patients) were reported. Out of 74 patients treated with 177Lu-DOTA-TATE, 5 cases of CR, 26 cases of PR, 14 cases of SD, and 29 cases of PD were observed based on biochemical response criteria. Moreover, 9 cases of PR, 50 cases of SD, and 26 cases of PD were achieved in 85 patients based on radiological response criteria. Furthermore, SD was found in 3 patients based on metabolic response criteria. In the patients treated with 111_Indium -based agent; 1 case of CR, 2 cases of SD, and 4 cases of PD (in 7 patients assessed based on biochemical response) and also, 2 cases of CR, 4 cases of SD, and 5 cases of PD (in 11 patients assessed based on radiological criteria) were reported.

Overall, in the patients with metastatic MTC, biochemical and objective responses were observed in 37.2 and 10.6% of the patients, respectively.

### Safety of PRRT

Safety of PRRT was assessed in 19 studies (totally, 239 patients). Death was observed in 109 patients. In addition, time to death varied from 1 to 63months.

In 95 patients with advanced RR-DTC, 46 patients died. Time to death ranged from 1 to 63months from commencement of PRRT. Based on type of PRRT, death occurred in 29/55 patients treated with ^90^Y -based agent, 6/17 patients treated with 177Lu-DOTA-TATE, and 8/16 patients treated with 111In-Octreotide. Among 44 patients with metastatic MTC, 63 patients died. Time to death ranged from 1 to 26.8months since initiating the first course of PRRT. Based on the type of PRRT, death occurred in 27/69 patients treated with ^90^Y-DOTATOC, 31/63 patients treated with 177Lu-DOTA-TATE, and 4/5 patients treated with 111In- Octreotide. Major side effects were reported in 124 patients treated with ^90^Y -based agent. Fourteen patients developed renal toxicity (2 cases of grade 4, 2 cases of grade 3, 2 cases of grade 2, and 8 cases of grade 1). Furthermore, hematologic toxicity was observed in 64 patients (3 cases developed grade 4 of thrombocytopenia, and 1 patient reported to suffer from grade 4 of anemia). Moreover, in 80 patients treated with 177Lu-DOTA-TATE, mild and transient hematologic and renal complications were reported (4 patients with grade 1 and one case with grade 2 of hematologic toxicity and one patient with grade 2 of renal toxicity). Among 21 patients treated with 111In- Octreotide, one patient developed transient thrombocytopenia (grade1).

## Discussion

Herein, a comprehensive systematic review was done to investigate efficacy and safety of PRRT in management of advanced RR-DTC and metastatic MTC. The results suggested that PRRT could maintain disease stability with few adverse events. In short-term, toxicity is mild and transient. In addition, long-term toxicity is rare and with low grade. To the best of our knowledge, no similar systematic review or meta-analysis has been done previously to investigate efficacy and safety of PRRT in RR-DTC and metastatic MTC.

There are few recommended treatments for the patients with RR-DTC and therapeutic options are associated with certain limitations in case of the patients with metastatic DTC. The choice of treatment depends on bulk of the tumor. Simple observation, multi-targeted, or mutation-selected kinase inhibitors (MKI), and traditional cytotoxic chemotherapy are the available options [12, 62]. Despite approval of doxorubicin by the food and drug administration (FDA), treatment with cytotoxic agents has shown disappointing results [63]. Therefore, benefit-risk ratio must be carefully evaluated before starting treatment [62].

For majority of the patients with MTC, primary surgery is curative at early stages. However, local and distant metastases after surgery are the major causes of mortality [14]. Resurgery, chemotherapy, external beam radiation therapy, and biological agents, such as RET and MEK inhibitors have yielded disappointing and limited results. Although, treatment with tyrosine kinase inhibitors (TKIs) (Vandetanib and Cabozantinib) improves progression-free survival (PFS), severe adverse events could limit the use of them. There is no curative treatment for these patients, and all the available treatment modalities have been shown to have certain limitations and complications [6].

In the 1990s, the role of SSTR in regulation and proliferation of normal thyroid cells and tumoral tissues was reported that led to introduction of peptide receptor imaging and PRRT in management of metastatic MTC and advanced RR-DTC [15]. Type of SSTRs expression could have an effect on survival rate of these patients [64]. From 5 subtypes of SSTR described in human cells, SSRT2 is expressed in MTC [7]. However, SSRT2 expression has not been identified in papillary or follicular thyroid cancer, and it is irregularly expressed in Hurthle cell adenoma and Hurthle cell carcinoma [65].

Generally, PRRT is able to deliver a high dose of radiation to intracellular components of cancer cells, and induce tumor shrinkage [7]. Currently, PRRT is considered as a safe and effective treatment modality for metastatic inoperable well-differentiated neuroendocrine tumors and advanced pheochromocytomas and paragangliomas [16, 17].

The most frequently used radionuclides in PRRT are ^90^Y and Lutetium-177. They have different physical characteristics, namely different emission ranges. This results in various maximum tissue penetrations ranging from 3mm for Lutetium-177 to 12mm for ^90^Y. Since, ^90^Y has the highest energy and maximum tissue penetration; it is a preferable radionuclide for tumors with large size and poor vascularization. On the other hand, Lutetium-177 emits intermediate-energy suitable for small-sized tumors. Few studies had used 111In-Octreotide, with tissue penetration ranging from 0.2 to 10mm (Table 1) [7]. Krenning et al., for the first time reported treatment of the patients with advanced DTC with 111In-Octreotide analogs. One patient, who received total cumulative activity of at least 20GBq showed disease stabilization [40]. In a pilot study conducted in Netherlands, 9 patients with advanced RR-DTC were treated with high, fixed doses of 111In -Octreotide. Six months after the last therapy, 4 patients had SD, and 5 patients showed PD. Mean Tg value was higher in PD cases than patients with SD. They concluded low Tg value could have a positive effect on the outcome [41].

Grges et al., in a study regarding the first cases of treatment with ^90^Y-DOTATOC in 3 patients with advanced RR-DTC and pulmonary metastasis showed deceleration in short-term disease progression [26]. In the last report on treatment with ^90^Y-DOTATOC in RR-DTC, median survival was found to be 21months from initiating the first course of PRRT with only minor and transient hematological toxicity in some patients [15]. Recently, 177Lu-DOTA-TATE has been used more than ^90^Y but, number of patients treated with this somatostatin analog was limited.

In the patients with metastatic MTC, limited experience with PRRT treatment has been reported. Results of a study on the patients with metastatic MTC suggested that treatment with ^90^Y-DOTATOC is associated with a long-term survival benefit. However, treatment response was independent of pre-treatment scintigraphy results [45]. Recently, Beukhof et al., reported 17years of experiences with 177Lu-octreotate treatment. They concluded that this treatment could be considered as a treatment in the patients with high uptake on 111In-DTPA-Octreotide scan (uptake grade 3) and positive SSTR2a expression in tumor histology [54]. Budiawan et al., found that the patients with RR-DTC having good response had less undergone other treatment modalities prior to PRRT than non-responders. In addition, they introduced lung metastasis as a poor prognostic factor for survival after PRRT [6].

However, PRRT is not free from adverse effects and minor complications,such as nausea, asthenia, and elevation in liver enzyme level are observed in up to 16.7% of patients, while major complications,such as nephrotoxicity and hematologic adverse events are rare and transient [23, 24]. Proximal tubular reabsorption of radio peptide and its interstitial retention lead to glomerular fibrosis [40], which is markedly observed after treatment with ^90^Y-DOTATOC. Hence, kidney protection is mandatory along with co-administration of positively-charged amino acids,such as L-lysine and/or L-arginine competitively inhibiting proximal tubular reabsorption of the radio peptide, or prolonged infusion over 10h to 2days after administration of radio peptide. Despite kidney protection, loss of renal function may become clinically evident years after PRRT, especially after administration of ^90^Y- DOTATOC. Sporadic reported cases of delayed renal failure have received activities greater than 7.4GBq /m2 in very few cycles, without kidney protection [61]. Cumulative and per-cycle renal uptake dose, age, hypertension, diabetes and previous chemotherapy with nephrotoxic agents could accelerate the decrease in renal function after PRRT [66]. Considering these risk factors, one can modify treatment plan or change choice of radio peptide based on burden of tumors. Hematologic side effects generally are mild and temporary,such as reduction in count of lymphocytes and platelets [57].

Our systematic review demonstrated that treatment with PRRT not only could lead to minor complications in approximately 10% of cases but also it can cause very rare and transient major complications.

This systematic review benefited from a comprehensive search conducted by two independent investigators, no time limits, independent reviews by two reviewers, and no publication bias. However, the main limitation of the present study was low quality of the available evidence. However, other underlying problems and limitations included retrospective nature of the studies, a selection bias, the amount of radioactivity administered (183GBq), non-uniform response criteria, huge difference in follow-up periods (199months),and the limited number of patients per report. Also, our search was restricted to English -language papers.

This systematic review investigated efficacy and safety of PRRT in treatment of RR-DTC and metastatic MTC. Given paucity of evidence, it is recommended to perform further multi-center randomized controlled clinical trials.

## Conclusions

According to findings of our study, due to lack of various treatment modalities, PRRT could be an option for treatment of advanced RR-DTC, as well as metastatic MTC, with few adverse events.

## Supplementary Information

**Additional file 1: Supplemental Table1.** Medline (Pubmed, Ovid and Ebsco), Scopus, Embase, Web of Science and the Cochrane Library database (Last Updated March 24, 2021).

**Additional file 2: Supplemental Table2.** Risk of bias assessment.

## Data Availability

All data generated or analyzed during this study are included in this published article [and its supplementary information files].

## Abbreviations

RR-DTC: Radioiodine-refractory differentiated thyroid cancer
MTC: Medullary thyroid cancer
PFS: Progression-free survival
SSTR: Somatostatin receptor
PRRT: Peptide receptor radionuclide therapy
TTP: Time to progression
Tg: Thyroglobulin
CEA: Carcino embryogenic antigen
WHO: World health organization
RECIST: Response evaluation criteria in solid tumors
SWOG: Southwest oncology group
EORTC: European organization for research and treatment of cancer
RAI: Radioactive iodine
CR: Complete response
SD: Stable disease
PR: Partial response
PD: Persistent disease
TKI: Tyrosine kinase inhibitors
MKI: Mutation-selected kinase inhibitors
FDA: Food and drug administration
