# Supplementary material for: Efficacy and safety of peptide receptor radionuclide therapy in advanced radioiodine-refractory differentiated thyroid cancer and metastatic medullary thyroid cancer: a systematic review
Source: BMC Cancer. 2021 May 20;21:579. doi: 10.1186/s12885-021-08257-x (PMC8139052; doi:10.1186/s12885-021-08257-x)
Supplement: Supplementary file 1 — Additional file 1: Supplemental Table1. Medline (Pubmed, Ovid and Ebsco), Scopus, Embase, Web of Science and the Cochrane Library database (Last Updated March 24, 2021). [file 12885_2021_8257_MOESM1_ESM.docx]

| **Supplemental table 1,**Medline (Pubmed, Ovid, Ebsco), Scopus, Embase, Web of Science and the Cochrane Library database (Last Updated March 24, 2021) |
| --- |
| **PubMed:**  (Peptide receptor radionuclide therapy[Tiab] OR Radionuclide therapy[Tiab] OR radiolabeled somatostatin analogues[Tiab] OR receptors peptide radioisotopes therapy[Tiab] OR PRRT[Tiab]) AND (thyroid cancer[Tiab] OR thyroid carcinoma[Tiab] OR differentiated thyroid cancer[Tiab] OR differentiated thyroid carcinoma[Tiab] OR medullary thyroid cancer[Tiab] OR medullary thyroid carcinoma[Tiab] OR "Thyroid Neoplasms"[Mesh] OR "Thyroid Cancer, Papillary"[Mesh] OR "Thyroid cancer, Hurthle cell"[Supplementary Concept] OR "Thyroid cancer, medullary"[Supplementary Concept] OR "Familial medullary thyroid carcinoma"[Supplementary Concept] OR "Thyroid cancer, follicular"[Supplementary Concept]) |
| **OVID:**  Peptide receptor radionuclide therapy[mp=ti,ab] OR PRRT[mp=ti,ab] OR Radionuclide therapy[mp=ti,ab] OR radiolabeled somatostatin analogues[mp=ti,ab] AND thyroid cancer[mp=ti,ab] OR thyroid carcinoma[mp=ti,ab] OR thyroid neoplasm*[mp=ti,ab] OR differentiated thyroid cancer[mp=ti,ab] OR differentiated thyroid carcinoma[mp=ti,ab] OR differentiated thyroid neoplasm [mp=ti,ab] OR medullary thyroid cancer[mp=ti,ab] OR medullary thyroid carcinoma[mp=ti,ab] OR medullary thyroid neoplasm*[mp=ti,ab] |
| **Ebsco:**  Peptide receptor radionuclide therapy OR PRRT OR Radionuclide therapy OR radiolabeled somatostatin analogues AND thyroid cancer OR thyroid carcinoma OR thyroid neoplasm* OR differentiated thyroid cancer OR differentiated thyroid carcinoma OR differentiated thyroid Neoplasm* OR medullary thyroid cancer OR medullary thyroid carcinoma OR medullary thyroid neoplasm* |
| **Scopus:**  (TITLE-ABS (Peptide receptor radionuclide therapy) OR TITLE-ABS (PRRT) OR TITLE-ABS (Radionuclide therapy) OR TITLE-ABS (radiolabeled somatostatin analogues) OR TITLE-ABS (radiolabeled somatostatin analogues)) AND (TITLE-ABS (thyroid cancer) OR TITLE-ABS (thyroid carcinoma) OR TITLE-ABS (thyroid neoplasm*) OR TITLE-ABS (differentiated thyroid cancer) OR TITLE-ABS (differentiated thyroid neoplasm) OR TITLE-ABS (differentiated thyroid carcinoma) OR TITLE-ABS (medullary thyroid cancer) OR TITLE-ABS (medullary thyroid carcinoma) OR TITLE-ABS (medullary thyroid neoplasm*)) |
| **Embase:**  (“Peptide receptor radionuclide therapy”:ab,ti OR “PRRT”:ab,ti OR “Radionuclide therapy”:ab,ti OR “radiolabeled somatostatin analogues”:ab,ti) AND (“thyroid cancer”:ab,ti OR “thyroid carcinoma”:ab,ti OR “thyroid neoplasm*”:ab,ti OR “differentiated thyroid cancer”:ab,ti OR “differentiated thyroid carcinoma”:ab,ti OR “differentiated thyroid neoplasm”:ab,ti OR “medullary thyroid cancer”:ab,ti OR “medullary thyroid carcinoma”:ab,ti OR “medullary thyroid neoplasm*”:ab,ti) |
| **Web of science:**  (TS=Peptide receptor radionuclide therapy OR TS=PRRT OR TS=Radionuclide therapy OR TS=radiolabeled somatostatin analogues) AND (TS=thyroid cancer OR TS=thyroid carcinoma OR TS=thyroid neoplasm* OR TS=differentiated thyroid cancer OR TS=differentiated thyroid carcinoma OR TS=differentiated thyroid neoplasm* OR TS=medullary thyroid cancer OR TS=medullary thyroid carcinoma OR TS=medullary thyroid neoplasm*) |
| **Cochrane:**  ("Peptide receptor radionuclide therapy"[ti,ab,kw] OR “PRRT"[ti,ab,kw] OR “Radionuclide therapy"[ti,ab,kw] OR “radiolabeled somatostatin analogues”[ti,ab,kw]) AND ("thyroid cancer"[ti,ab,kw] OR "thyroid carcinoma"[ti,ab,kw] OR "thyroid neoplasm*"[ti,ab,kw] OR "differentiated thyroid cancer"[ti,ab,kw] OR "differentiated thyroid carcinoma"[ti,ab,kw] OR "differentiated thyroid neoplasm"[ti,ab,kw] OR " medullary thyroid cancer"[ti,ab,kw] OR "medullary thyroid carcinoma"[ti,ab,kw] OR "medullary thyroid neoplasm*"[ti,ab,kw]) |
| **Google Scholar search strategy Keywords include:**  "Peptide receptor radionuclide therapy", "PRRT", "Radionuclide therapy", “radiolabeled somatostatin analogues ", " thyroid cancer ", "thyroid carcinoma”, "thyroid neoplasm”, "differentiated thyroid cancer", "differentiated thyroid carcinoma", "differentiated thyroid neoplasm", " medullary thyroid cancer”, "medullary thyroid carcinoma”, "medullary thyroid neoplasm*" |
