# Supplementary material for: Efficacy and safety of peptide receptor radionuclide therapy in advanced radioiodine-refractory differentiated thyroid cancer and metastatic medullary thyroid cancer: a systematic review
Source: BMC Cancer. 2021 May 20;21:579. doi: 10.1186/s12885-021-08257-x (PMC8139052; doi:10.1186/s12885-021-08257-x)
Supplement: Supplementary file 2 — Additional file 2: Supplemental Table2. Risk of bias assessment. [file 12885_2021_8257_MOESM2_ESM.docx]

| **Supplemental table 2. Risk of bias assessment** | | | | | |
| --- | --- | --- | --- | --- | --- |
| **Author and publication year (citation)** | **Selection** | **Ascertainment** | **Causality** | **Reporting** | **Risk of bias judgement** |
| Basu, S et al. 2020([37](#_ENREF_37)) | Satisfy | Reliable | Strong | Adequately | Low |
| Bertagna F et al. 2009 ([38](#_ENREF_38)) | Possible selection bias | Reliable | Strong | Adequately | Low |
| Beukhof, C et al. 2019 ([31](#_ENREF_31)) | Satisfy | Reliable | Strong | Inadequately | Moderate |
| Bilgic, S. et al 2020 ([39](#_ENREF_39)) | Possible selection bias | Reliable | Strong | Inadequately | Moderate |
| Bodei L et al. 2008 ([34](#_ENREF_34)) | Satisfy | Reliable | Strong | Adequately | Low |
| Bodei L et al. 2004 ([40](#_ENREF_40)) | Satisfy | Reliable | Strong | Adequately | Low |
| Budiawan H et al. 2013 ([6](#_ENREF_6)) | Satisfy | Reliable | Strong | Inadequately | Moderate |
| Buscombe JR et al. 2003 ([41](#_ENREF_41)) | Satisfy | Reliable | Strong | Adequately | Low |
| Campenni A et al. 2015 ([42](#_ENREF_42)) | Possible selection bias | Reliable | Strong | Adequately | Low |
| Caplin M et al. 2000 ([36](#_ENREF_36)) | Possible selection bias | Reliable | Weak | Inadequately | High |
| Cinkir, H. Y et al. 2020 ([43](#_ENREF_43)) | Satisfy | Reliable | Strong | Adequately | Low |
| Czepczynski R et al. 2014 ([15](#_ENREF_15)) | Satisfy | Reliable | Strong | Adequately | Low |
| Elboğa, U et al. 2016 ([44](#_ENREF_44)) | Possible selection bias | Reliable | Strong | Inadequately | Moderate |
| Gabriel M et al. 2004 ([45](#_ENREF_45)) | Possible selection bias | Reliable | Strong | Inadequately | Moderate |
| Gao ZR et al. 2004 ([46](#_ENREF_46)) | Possible selection bias | Reliable | Strong | Adequately | Low |
| Gorges R et al. 2001 ([29](#_ENREF_29)) | Satisfy | Reliable | Strong | Adequately | Low |
| Hayes AR et al. 2019 ([47](#_ENREF_47)) | Possible selection bias | Reliable | Strong | Inadequately | Moderate |
| Iten F et al. 2009 ([33](#_ENREF_33)) | Satisfy | Reliable | Strong | Inadequately | Moderate |
| Iten F et al. 2007 ([30](#_ENREF_30)) | Satisfy | Unreliable | Strong | Inadequately | Moderate |
| Jois B et al. 2014 ([48](#_ENREF_48)) | Satisfy | Reliable | Strong | Inadequately | Moderate |
| Krenning E et al. 1999 ([27](#_ENREF_27)) | Satisfy | Reliable | Strong | Inadequately | Moderate |
| Makis W et al. 2015 ([49](#_ENREF_49)) | Possible selection bias | Reliable | Strong | Adequately | Low |
| Mathew, D et al. 2018 ([50](#_ENREF_50)) | Satisfy | Reliable | Weak | Inadequately | Moderate |
| Öksüz M et al. 2014 ([51](#_ENREF_51)) | Satisfy | Reliable | Strong | Inadequately | Moderate |
| Oliván-Sasot .P et al. 2017 ([52](#_ENREF_52)) | Possible selection bias | Reliable | Strong | Adequately | Low |
| Otte A et al. 1999 ([53](#_ENREF_53)) | Possible selection bias | Reliable | Strong | Adequately | Low |
| Parihar AS et al.2018 ([54](#_ENREF_54)) | Possible selection bias | Reliable | Weak | Adequately | Moderate |
| Pasieka JL et al. 2004 ([55](#_ENREF_55)) | Satisfy | Reliable | Strong | Adequately | Low |
| Parghane, R. V et al. 2020([56](#_ENREF_56)) | Satisfy | Reliable | Strong | Adequately | Low |
| Puranik A et al. 2019 ([57](#_ENREF_57)) | Satisfy | Unreliable | Strong | Inadequately | Moderate |
| Roll. W et al. 2018 ([58](#_ENREF_58)) | Satisfy | Reliable | Strong | Adequately | Low |
| Scalorbi F et al. 2017 ([59](#_ENREF_59)) | Satisfy | Unreliable | Weak | Inadequately | High |
| Soydal Ç et al. 2014 ([60](#_ENREF_60)) | Satisfy | Reliable | Strong | Adequately | Low |
| Stokkel MP et al. 2004 ([28](#_ENREF_28)) | Satisfy | Reliable | Strong | Adequately | Low |
| Teunissen JJ et al. 2005 ([61](#_ENREF_61)) | Satisfy | Reliable | Strong | Adequately | Low |
| Traub-Weidinger T et al. 2011 ([62](#_ENREF_62)) | Possible selection bias | Reliable | Strong | Inadequately | Moderate |
| Vaisman F et al. 2015 ([63](#_ENREF_63)) | Satisfy | Reliable | Strong | Adequately | Low |
| Valkema R et al. 2002 ([64](#_ENREF_64)) | Satisfy | Reliable | Strong | Adequately | Low |
| Versari et al. 2014([32](#_ENREF_32)) | Satisfy | Reliable | Strong | Adequately | Low |
| Virgolini I et al. 2002 ([65](#_ENREF_65)) | Satisfy | Reliable | Strong | Inadequately | Moderate |
| Waldherr C et al. 2001 ([66](#_ENREF_66)) | Possible selection bias | Reliable | Strong | Adequately | Low |
